# Supplementary material for: Biogeochemical Factors of Cs, Sr, U, Pu Immobilization in Bottom Sediments of the Upa River, Located in the Zone of Chernobyl Accident
Source: Biology (Basel). 2022 Dec 21;12(1):10. doi: 10.3390/biology12010010 (PMC9854679; doi:10.3390/biology12010010)
Supplement: Supplementary file 1 [file biology-12-00010-s001.zip › biology-2062330-supplementary.pdf]

Supplimentary files

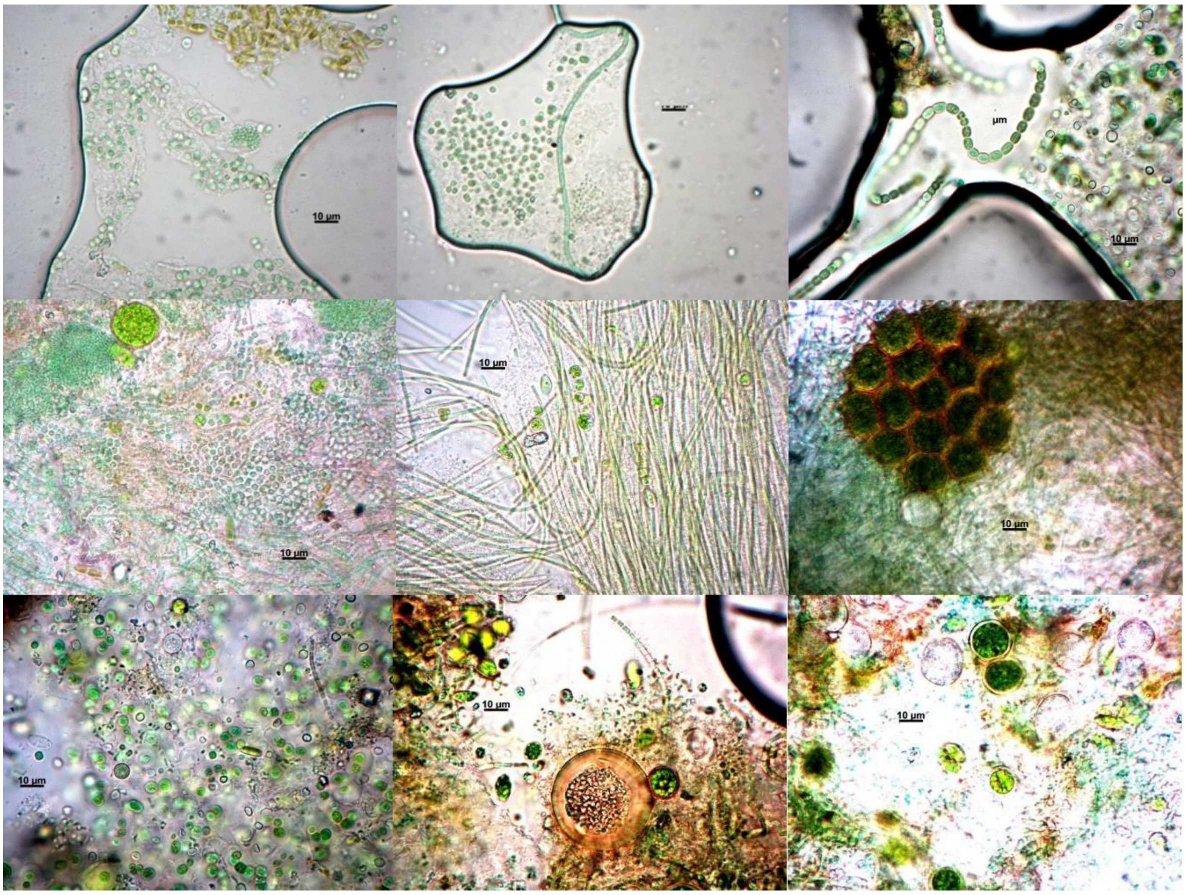

Figure S1. Phytoplankton diversity in a water sample from the Upa River.

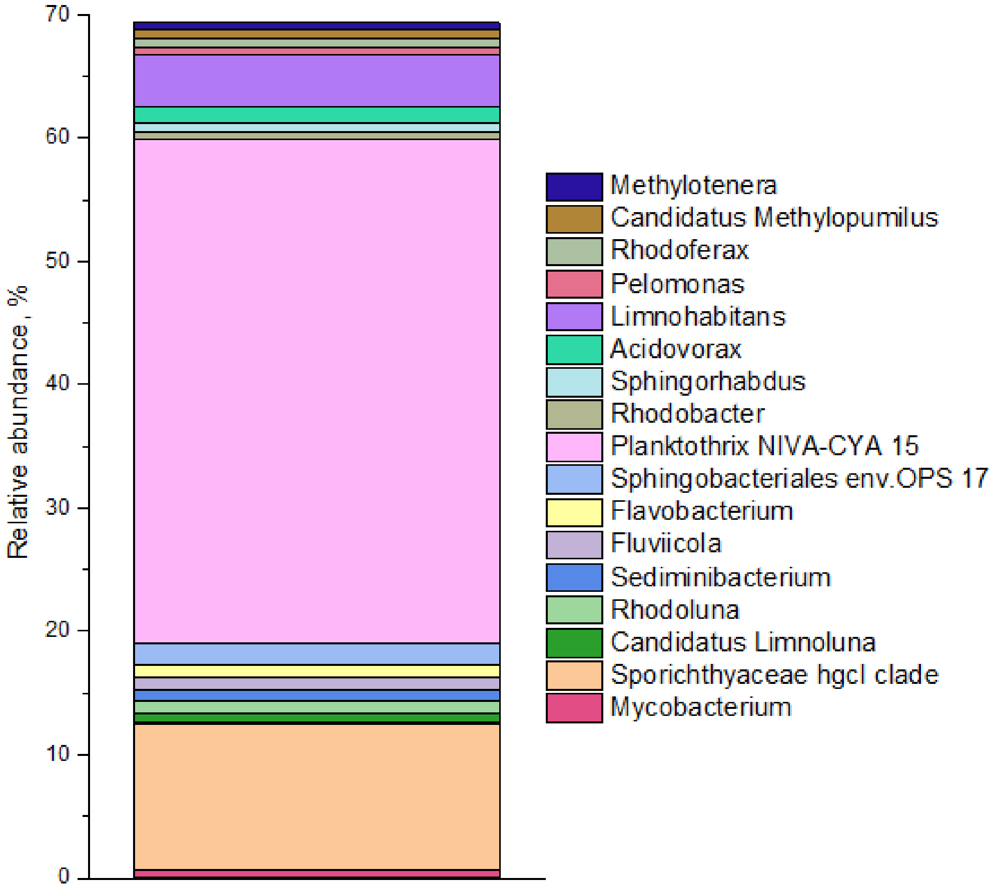

Figure S2. Prokaryotic community diversity profile of the phytoplankton community by 16S rNA genes.

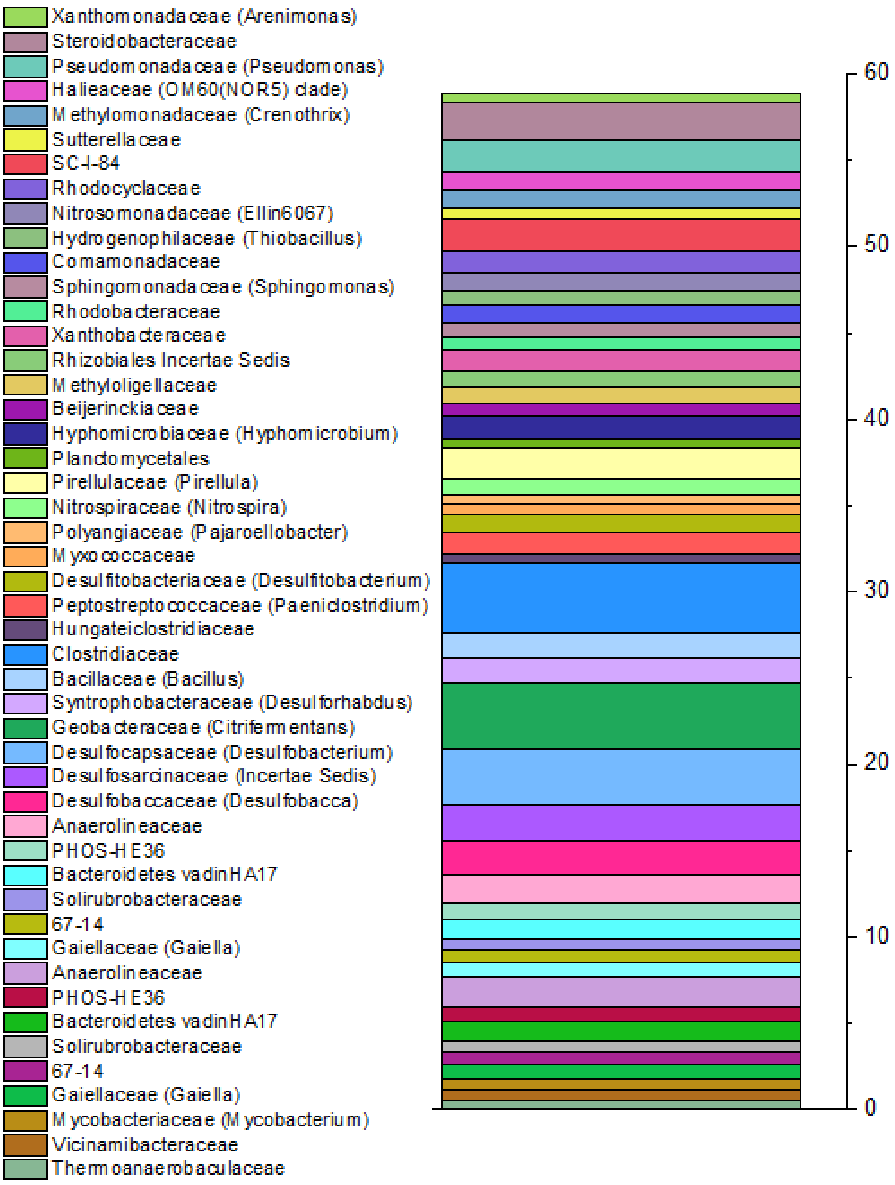

Figure S3. Profiling the microbial community of an anaerobic sludge sample by 16S rRNA genes.
